# Environmental Consequences of Invasive Species: Greenhouse Gas Emissions of Insecticide Use and the Role of Biological Control in Reducing Emissions

**DOI:** 10.1371/journal.pone.0072293

**Published:** 2013-08-20

**Authors:** George E. Heimpel, Yi Yang, Jason D. Hill, David W. Ragsdale

**Affiliations:** 1 Department of Entomology, University of Minnesota, St. Paul, Minnesota, United States of America; 2 Bren School of Environmental Science and Management, University of California Santa Barbara, Santa Bárbara, California, United States of America; 3 Department of Bioproducts and Biosystems Engineering, University of Minnesota, St. Paul, Minnesota, United States of America; 4 Department of Entomology, Texas A&M University, College Station, Texas, United States of America; U. Kentucky, United States of America

## Abstract

Greenhouse gas emissions associated with pesticide applications against invasive species constitute an environmental cost of species invasions that has remained largely unrecognized. Here we calculate greenhouse gas emissions associated with the invasion of an agricultural pest from Asia to North America. The soybean aphid, *Aphis glycines*, was first discovered in North America in 2000, and has led to a substantial increase in insecticide use in soybeans. We estimate that the manufacture, transport, and application of insecticides against soybean aphid results in approximately 10.6 kg of carbon dioxide (CO_2_) equivalent greenhouse gasses being emitted per hectare of soybeans treated. Given the acreage sprayed, this has led to annual emissions of between 6 and 40 million kg of CO_2_ equivalent greenhouse gasses in the United States since the invasion of soybean aphid, depending on pest population size. Emissions would be higher were it not for the development of a threshold aphid density below which farmers are advised not to spray. Without a threshold, farmers tend to spray preemptively and the threshold allows farmers to take advantage of naturally occurring biological control of the soybean aphid, which can be substantial. We find that adoption of the soybean aphid economic threshold can lead to emission reductions of approximately 300 million kg of CO_2_ equivalent greenhouse gases per year in the United States. Previous studies have documented that biological control agents such as lady beetles are capable of suppressing aphid densities below this threshold in over half of the soybean acreage in the U.S. Given the acreages involved this suggests that biological control results in annual emission reductions of over 200 million kg of CO_2_ equivalents. These analyses show how interactions between invasive species and organisms that suppress them can interact to affect greenhouse gas emissions.

## Introduction

Many pest organisms reach their most damaging levels away from their native geographic range. This general pattern has been documented for weeds [1], insect pests [2], and pathogens [3,4] among others. Extensive bodies of literature have developed around both the causes and consequences of invasive pests [5–7]. Although invasive species can cause considerable economic and ecological damage [8–10], biotic resistance by competitors and consumers of introduced species can greatly attenuate this damage (e.g. [11–13]). Here, we consider the implications of an invasive agricultural pest for greenhouse gas emissions associated with controlling the pest. Our goal is to estimate the actual emission costs incurred by the invasion as well as the hypothetical emissions that would occur in the absence of biotic resistance in the form of naturally occurring biological control. This allows an estimation of the role of biological control in attenuating greenhouse gas emissions of an invasive pest.

Numerous recent analyses link global climate change to invasive species (e.g. [14–16]). Most of these analyses focus on effects of climate change on the spread or consequences of invasive species, but the converse of this relationship - effects of invasive species on climate change – remains virtually unexplored. The only such analysis of which we are aware is the one by Kurz et al. [17], which showed how climate change-induced spread of bark beetles in Canada can lead to reduced carbon sequestration. Our analysis highlights another effect of invasive species on climate change: impacts of invasive species on greenhouse gas emissions. By virtue of their increased abundance in introduced ranges, invasive pests require disproportionately high levels of management intervention, including efforts at eradication and control [8]. We focus on greenhouse gas emissions associated with insecticide use to control the soybean aphid, *Aphis glycines*, a recent invasive agricultural pest in North America.

The soybean aphid is native to eastern Asia and was first detected in North America in the summer of 2000. Although insect predators are important in reducing the damaging effects of the soybean aphid, this insect has emerged as the most important pest of soybeans in North America [18]. Management of the soybean aphid has been primarily through application of insecticides although alternative management tactics including host-plant resistance and the importation of Asian biological control agents are also under development [18–20].

Our aim in this paper is to calculate the life cycle greenhouse gas emissions associated with insecticide use against the soybean aphid in the United States, taking into account insecticide manufacture, transport, and application. Other researchers have prepared energy budgets and have estimated greenhouse emissions for various agricultural practices including pesticide use [21–25], but to our knowledge this is the first analysis focused on a particular pest species. We also consider the extent to which economic spray thresholds [26,27] and naturally occurring biological control (e.g. [28–31]) can mitigate carbon emissions associated with soybean aphid control.

## Analysis and Results

Our estimate of life cycle greenhouse gas emissions induced by chemical control of soybean is divided into emissions associated with insecticide manufacture, transport, and application. We use estimates of insecticide application associated with soybean aphid control from the United States Department of Agriculture National Agricultural Statistics Service (USDA NASS) Agricultural Chemical Usage Field Crops Summary databases. These databases provide state-level data on quantities applied (L ha^-1^) and acreage (ha) treated and are available for insecticide use in soybeans from 1991 through 2002 and from 2004 through 2006. We focus exclusively on the 12 states within the North-central region of the U.S., as defined by USDA NASS – Illinois, Indiana, Iowa, Kansas, Michigan, Minnesota, Missouri, Nebraska, North Dakota, Ohio, South Dakota and Wisconsin. The rationale for not including data from other states is that farmers in some other states, particular in the southeastern U.S., apply insecticides against insect pests other than the soybean aphid [32–34]. Soybean acreage in the North-Central states, on the other hand, received almost no insecticide application prior to the arrival of soybean aphid. It is therefore likely that most if not all of the application of insecticides in these states following the arrival of soybean aphid was directed at soybean aphid [18,26]. The 12 states used in our analysis cover approximately 80% of the approximately 38 million hectares of soybeans planted each year in the U.S.

### Manufacture

**Figure 1:**
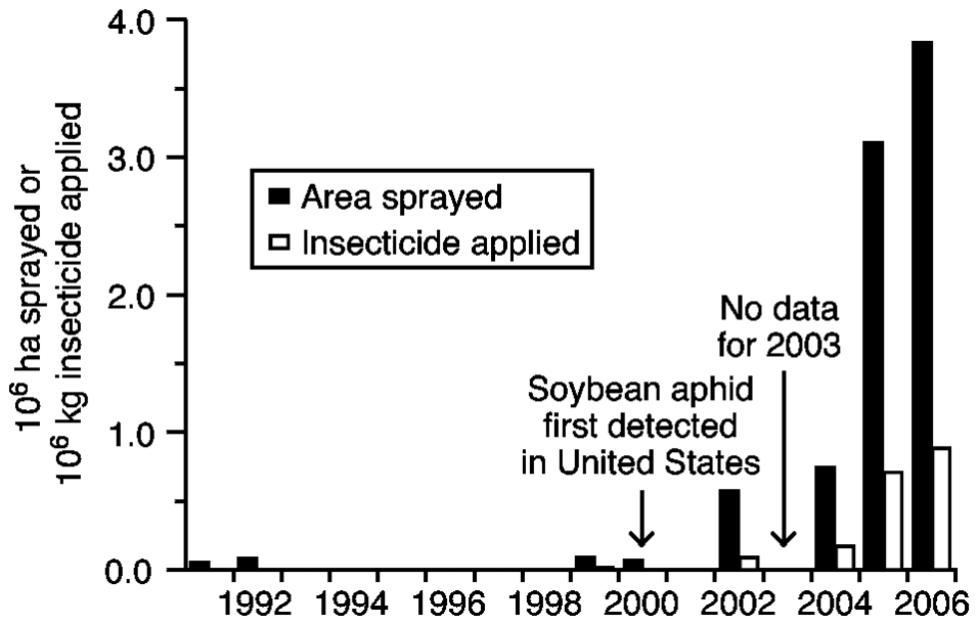
Millions of kilograms of insecticide (grey bars) and millions of hectares onto which insecticides were sprayed (black bars) at least once in 12 North-central states in the U.S. (IL, IN, IA, KS, MI, MN, MO, NE, ND, OH, SD, WI) between 1991 and 2006. Data are from the U.S. Department of Agriculture National Agricultural Service, from which data on insecticide use in soybeans were available for the years 1991-2002 and 2004-2006.

We used information on the amount of active ingredient (A.I.) of insecticide applied per year against soybean aphid to estimate emissions associated with insecticide manufacture and transport. The amounts of insecticide applied were too low to be reported by USDA NASS between 1991 and 1998 although some acreage was reported treated in 1991 and 1992 (Figure 1). In 1999 – the year before soybean aphid was detected in North America -15,400 kg of insecticides were applied to soybeans in North-Central states against other insects. The figure was about one-third of this amount in 2000 but then increased to almost 0.9 million kg by 2006 (Figure 1).

The main insecticides used against soybean aphid were chlorpyrifos (an organophosphate), lambda-cyhalothrin, and esfenvalerate (both pyrethroids) with lesser use of zeta-cypermethrin and permethrin (both pyrethroids as well). The relative percentages of chlorpyrifos, lambda-cyhalothrin, and esfenvalerate used over the entire reporting period were 38%, 47%, and 15%, respectively, according to the USDA NASS database. All three compounds are broad-spectrum insecticides that exhibit high toxicity to honeybees, other beneficial insects including biological control agents of aphids, and vertebrates (e.g. [35–37]). The neonicotinoid class of insecticides are used as a seed treatment targeting soybean aphid as well [38,39] but these compounds are not tracked by USDA NASS, so we do not consider them here.

Energy inputs associated with insecticide manufacture include the raw materials themselves, which are typically petroleum or natural gas, and the transformation of these materials into insecticides using a variety of energy-intensive industrial processes [25]. A life cycle analysis also includes energy inputs needed to build manufacturing plants and related operations, as well as those needed to extract the fuel needed for manufacture. Green [25] was the first to construct life cycle energy budgets for pesticide manufacture with these principles in mind and his analyses, although only approximations, have been used by a number of authors and remain the most reliable estimates to date [40–42]. Green [25] produced estimates for 39 commonly used pesticides, 11 of which are insecticides. These compounds did not include the three foliar insecticides used against soybean aphid noted above, but other organophosphates and pyrethroids were represented. Our approach here is to use the information provided by Green [25] for insecticides belonging to the same class of compounds in our analysis of the manufacture of soybean aphid insecticides. Specifically, we use Green’s energy values for cypermethrin to approximate values for lambda cyhalothrin and esfenvalerate since all three are classified as fourth-generation pyrethroids [43] or as Type II pyrethroids based upon their chemical structure [44]. To approximate energy use associated with chlorpyrifos, we use Green’s values for methyl parathion since both insecticides are classified in the same subclass of organophosphate insecticides, the phosphorothiolates [44].

The estimate of energy inputs provided by Green [25] reported proportions of different energy and material inputs used to manufacture various pesticides. Here we convert Green’s estimates associated with manufacturing and transport to their greenhouse gas emissions factors. Green’s analyses reported energy use in joules, from which we derive estimates of CO_2_ equivalent (CO_2_e) greenhouse gas emissions based upon global warming potential. The three main greenhouse gasses are CO_2_, methane (CH_4_), and nitrous oxide (N_2_O), which have default standardized 100-year global warming potentials of 1, 25, and 298, respectively [45]. Further, Green included both process energy (fuel oil, electricity, and steam) used or combusted on-site, and inherent energy (naphtha, natural gas, and coke) used as material feedstock and these processes require different conversion factors to CO_2_e. For process-energy related greenhouse gas emissions, we applied the following emission conversion factors: 0.090 kg CO_2_e MJ^-1^ for fuel oil, 0.095 kg CO_2_e MJ^-1^ for steam generated from fuel oil and 0.772 kg CO_2_e kWh^-1^ for electricity. These emission factors were taken from the Ecoinvent v. 2.2 database [http://www.ecoinvent.ch/] reflecting US emissions. For feedstock-associated GHG emissions, we applied the following emission factors: 0.005 kg CO_2_e MJ^-1^ for naphtha, 0.016 kg CO_2_e MJ^-1^ for natural gas and 0.018 kg CO_2_e MJ^-1^ for coke (Greenhouse Gases, Regulated Emissions and Energy Use in Transportation Model (GREET); http://greet.es.anl.gov/). Finally, to estimate the eventual acreage onto which insecticides were applied in the field based on the documented manufacture rates (Figure 1), we used the application rates from insecticide labels to estimate the amount of active ingredient of each insecticide used per hectare.

The values and calculations outlined above are presented in Table 1 for the three foliar insecticides commonly used against soybean aphid. Based on the relative use of these insecticides, and their application rates, our calculations lead to an estimate of 6.93 kg of CO_2_e greenhouse gasses emitted per hectare sprayed. These analyses illustrate the differences in emissions associated with the different insecticide classes. For instance, while the manufacture of pyrethroids (such as lambda cyhalothrin and esfenvalerate) is more energy intensive than manufacture of organophosphates (such as chlorpyifos), the much lower application rate of pyrethroids [46] offsets this difference so that the pyrethroids result in lower per-hectare emissions (Table 1).

**Table 1:** Summary of life cycle analysis for greenhouse gas emissions associated with the manufacture and transportation of foliar insecticides against the soybean aphid in the United States.

Soybean aphid insecticide (relative use)	Corresponding compound	Total energy inputs (MJ/kg)^a^	kg CO_2_e/ kg A.I.: Manufacture	kg CO_2_e/kg A.I.: Transportation	Application rate (kg [A.I.]/Ha)	Kg CO_2_e/Ha: Manufacture	kg CO_2_ e/ha: Transport	kg CO_2_e/Ha: Manufacture + Transport
Lambda cyhalothrin (0.47)	Cypermethrin	580.000	65.165	0.082	0.022	1.434	0.002	**1.436**
Esfenvalerate (0.15)	Cypermethrin	580.000	65.165	0.082	0.045	2.934	0.004	**2.938**
Chlorpyrifos (0.38)	Methyl Parathion	160.000	18.198	0.082	0.841	15.306	0.069	**15.376**
**Totals weighted by relative use**		**420.400**	**47.340**	**0.082**		**6.931**	**0.028**	**6.958**

^a^ from Green (1987).

‘Corresponding compound’ refers to the insecticides most closely related to ones used against soybean aphid for which Green (1987) calculated energy inputs (see text).

### Transportation

Energy inputs associated with the transportation of insecticides from the manufacturing plant to the farm includes three steps: (1) transport from the plant to a distribution center, (2) transport from the center to a mixing facility where ingredients are combined, and (3) transport from the mixing facility to the farm. An analysis by Wang [47] estimated 837 km for the one-way distance for step (1) based upon the typical regional distribution of insecticide manufacturing plants and we use this estimate for our analysis. Wang further assumed that this transport would typically be achieved by barge or rail transport, which have very similar GHG emissions (0.046 and 0.050 kg CO_2_e per ton*km, respectively; Ecoinvent v. 2.2). Steps (2,3) were estimated to be 80 and 48 km respectively, with corresponding GHG emissions of 0.134 and 0.239 kg CO_2_e per ton*km respectively, based upon the type of trucks typically used for these steps (Ecoinvent v. 2.2). Total emissions associated with these transportation steps converted to CO_2_ equivalents per hectare eventually sprayed are shown in Table 1. The overall value, weighted by the insecticides used is just under 0.03 kg CO_2_e ha^-1^, thus bringing the emissions associated with both manufacture and transportation to 6.96 kg CO_2_e ha^-1^ (Table 1).

### Application

Greenhouse gas emissions associated with insecticide application are a function of the total acreage sprayed and the number of applications per year. In the 9 years prior to the arrival of soybean aphid, between 0 and 90,500 hectares of soybeans received insecticides in the North-Central U.S. (average between 1991 and 1999 = 24,700 hectares per year). Between 2000 and 2006, however, soybean acreage receiving insecticides increased to a maximum of 3.85 million hectares per year in 2006 (average between 2000 and 2006 excluding 2003, for which there is no data = 1.4 million hectares per year). The acreage sprayed in 2006 represented 19% of the total soybean acreage in the 12 North-Central states. According to USDA NASS, applications were made once per year in individual fields, with multiple applications rarely recorded. For simplicity, we therefore based our calculations on a single application per year.

Ground application was the dominant mode of insecticide application against soybean aphid [48] and many farmers in the region purchased specialized spray equipment for tractors in response to soybean aphid [26]. Furthermore, Helsel [23] noted that the energy used for ground and air application of pesticides is similar on large acreages. For simplicity we therefore base our calculations on ground application only.

To estimate emissions associated with ground application, we used values from the GREET database [24]. The GREET estimate of emissions due to application of any insecticide is 3.60 kg CO_2_e greenhouse gases per hectare, which is due to the use of diesel fuel, which is estimated at 1.2 liters per hectare for tractor-drawn sprayers [49]. The practice of ‘tank-mixing’ insecticides with herbicides, fertilizers and fungicides can lead to a lower level of applications dedicated to insecticide use. In one estimate, tank-mixing with the herbicide glyphosate accounted for nearly 40% of soybean aphid insecticide applications in 2007 [48], with much lower incidence of tank-mixing with fertilizer and undocumented levels of mixing with fungicide. However, these practices were not widely used during the time of our analyses [48] and they are not recommended because of reduced effectiveness of the insecticide [50].

**Figure 2:**
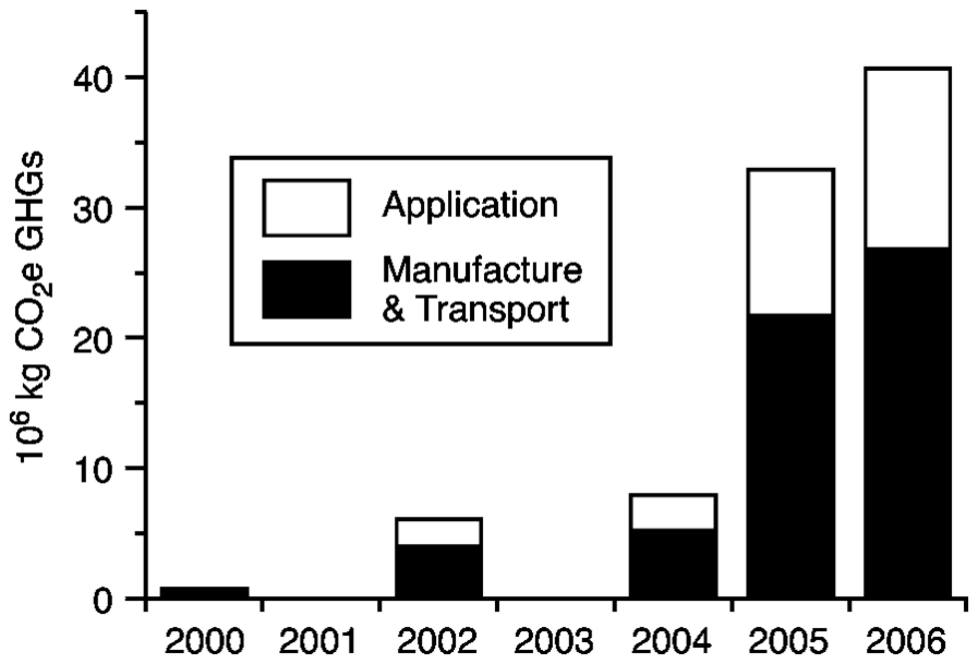
Estimates of emissions of CO_2_e greenhouse gases in millions of kilograms associated with the use of foliar insecticides against the soybean aphid in the United States. Estimates are provided for the years since soybean aphid was first detected in the U.S. and estimates of insecticide usage were reported by the United States Department of Agriculture National Agricultural Statistics Service (USDA NASS).

In Figure 2, we show the estimated total CO_2_ equivalent greenhouse gases emitted per year due to manufacture, transportation and application of active ingredients of foliar insecticide against soybean aphid based upon acreage treated from Figure 1. We estimated that the use of foliar insecticides against the soybean aphid in the United States leads to approximately 10.6 kg of CO_2_ equivalent GHGs emitted per hectare sprayed -7.0 kg from insecticide manufacture and transport, and 3.6 kg from the application process.

### Spray thresholds and biological control

A number of factors can mitigate insecticide use against soybean aphid, maintaining greenhouse gas emissions below what they otherwise would be. These include the development of a spray threshold for soybean aphid and the action of aphid consumers in suppressing soybean aphid below the threshold level. An economic spray threshold is the pest density at which a farmer must spray to avoid economic yield loss exceeding the cost of the application. In the absence of a threshold, farmers tend to spray on a schedule even when the pest is absent or at very low levels. For soybean aphid, a study done across the North-Central region of the U.S. calculated a threshold of 250 aphids per plant [26]. This threshold began to be disseminated in 2004 and was increasingly adopted throughout the region, replacing prophylactic treatment which leads to unnecessary application [27,48].

One of the main reasons that pest densities remain below threshold levels is that they are consumed by natural enemies. The role of naturally occurring predators and parasitoids in maintaining soybean aphid below the 250 per-plant threshold has been determined [30,51], and the extent to which these ‘biocontrol services’ lead to reductions in insecticide use has been calculated [52]. These analyses suggested that between 60 and 100% of soybean fields would exceed threshold levels in the absence of natural biological control and insecticide use, depending on aphid pressure in a particular year. In contrast, only 0-30% of fields actually exceeded threshold in the presence of aphid natural enemies, primarily lady beetles in these studies. This latter range of values encompasses the status quo and illustrates the value of the threshold over prophylactic treatments (i.e. preventative spraying in every field). Over the 30 million hectares of soybeans in the North-Central U.S., prophylactic spraying would result in emissions of 318 million kg CO_2_e greenhouse gases; full adoption of the threshold would reduce this by 70-100%.

Using a spray threshold allows for biological control to drastically reduce the need for insecticide applications. If we assume that 80% of the acreage would exceed threshold in the absence of aphid enemies in a typical year and 15% in the presence of enemies [52], CO_2_e greenhouse gas emissions would decrease from an estimated 254 million kg to 48 million kg per year within the North-Central U.S. (assuming again that 30 million hectares are planted to soybeans in this region). This implies that the action of natural enemies of aphids coupled with the use of a spray threshold reduces greenhouse gas emissions by approximately 207 million kg of CO_2_e per year.

## Discussion

We estimated emissions of 10.6 kg CO_2_ equivalent greenhouse gasses per hectare of soybeans sprayed with insecticides against the soybean aphid, an insect pest native to Asia that invaded North America in the year 2000. These values are similar to estimates for emissions associated with insecticide use by other authors [22,23]. Since the invasion of soybean aphid, insecticide use in soybeans in the North-Central United States has increased from less than 25,000 hectares per year prior to 2001 to levels approaching 4 million hectares in 2006. The USDA NASS database estimates that insecticides have been used against soybean aphid on a total of 8.32 million hectares during the years 2001, 2002, 2004, 2005, and 2006 (data for other years following the soybean aphid invasion are not available). Our estimate of GHG emissions attributable to insecticide use directed at soybean aphid for these years in the United States is therefore 87.2 million kg CO_2_e, or an average of 17.4 million kg CO_2_e greenhouse gases per year during this period. As a point of reference, this annual amount of emissions is equivalent to CO_2_e produced by the burning of approximately 7.4 million liters of gasoline, and could be offset by CO_2_ sequestration achieved by approximately 5,800 hectares of U.S. forest land per year (U.S. E. PA. Greenhouse Gas Equivalencies Calculator; updated Oct. 2012; http://www.epa.gov/cleanenergy/energy-resources/calculator.html).

The magnitude of these emissions would be far greater were it not for biological control of the soybean aphid, and an economic spray threshold that allows farmers to take advantage of biological control and other factors that limit soybean aphid populations. Using existing data [30,52] we estimated that the spray threshold reduced potential emissions as much as 300 million kg CO_2_e greenhouse gases per year and that reductions of emissions due to biological control maintaining aphid densities below the threshold at over 200 million kg CO_2_e. This amount of emissions reduction is equivalent burning approximately 88 million liters of gasoline or to sequestration achieved by 68,700 hectares of U.S. forest land (U.S. E.P.A. Greenhous Gas Equivalencies Calculator). While a number of economic and ecological benefits have been attributed to biological control [53–57], this is the first consideration of benefits accruing through reduction of greenhouse gas emissions.

Our estimate of emissions attributable to soybean aphid management is conservative for a number of reasons. First, our per-hectare estimates of insecticide manufacture underestimate emissions because only the active ingredient is included. Additives such as adjuvants are not included in the USDA NASS estimates of kg of insecticide use. We also do not include emissions associated with the use of insecticides applied as seed treatments, which are primarily the neonicotinoids. One estimate from 2010 was that 20% of soybean seeds on the market contained a neonicotinoid seed treatment, but this is increasing [58]. Greenhouse gas emissions associated with seed treatments are exclusively due to manufacturing costs because post-planting application is avoided, but our analysis showed that the energy required to manufacture insecticides can exceed the energy used to apply them. Another source of GHG emissions not included involves the manufacture and maintenance of specialized equipment for insecticide application against soybean aphid. It does appear that many growers purchased a sprayer specifically for use against soybean aphid [26] and the associated emissions could reasonably be added to our overall estimate. However, this equipment could have other uses, notably for fungicide applications [59], so we have omitted this source of emissions from our analysis. Lastly, due to data collection limitations our estimate of total emissions does not include the years 2003, 2007 or 2008, which were characterized by high soybean aphid densities in at least parts of the region [59,60].

Many pest management tactics that reduce the need to apply foliar insecticides have the potential to further reduce greenhouse gas emissions. Three tactics with potential for such effects are (i) host plant resistance, (ii) cultural control methods, and (iii) classical biological control. Soybean varieties that exhibit resistance to soybean aphid have been developed and have the potential to greatly reduce insecticide use [18]. A caveat is that strains (“biotypes”) of soybean aphids have been found that are able to develop normally on resistant soybean cultivars [20]. Cultural control methods against the soybean aphid are based on the observation that biological control can be enhanced by habitat diversification ( [30,61] but see 51,62) These methods can include cover cropping strategies that reduce pest pressure [63–65] and thus the need to apply insecticides, but may also increase some emissions through increased cultivation or planting. Classical biological control, in which exotic natural enemies of soybean aphid are imported from its native range could reduce or even eliminate the need to apply insecticides if successful [19]. To date however, no classical biological control has been successfully established [18,66,67].

We have used information on insecticide applications against the soybean aphid in the United States to show that invasive species can lead to significant greenhouse gas emissions that would not have occurred in the absence of the invasion. Invasive species are expected to have implications for the carbon cycle beyond pesticide use, however, and these include effects on other management strategies. For example, bacterial nitrogen fixation in soybeans is reduced by soybean aphid infestation [68], which could lead to increased nitrogen fertilizer inputs and attendant greenhouse gas emissions. Invasive species can also decrease carbon sequestration by plants as has been shown for bark beetles [17]. Other effects on sequestration are possible as well, depending upon the ecological effects of the invading species. Our analysis highlights the fact that invasive pest species are not only affected by global climate change, but they can also affect it – in this case by increasing the greenhouse gas emissions associated with management tactics.

## References

[1] Myers JH, Bazely DR (2003) Ecology and Control of Introduced Plants. Cambridge, UK: Cambridge University Press. 313pp.

[2] Kenis M, Auger-Rozenberg MA, Roques A, Timms L, Pere C et al. (2009) Ecological effects of invasive alien insects. Biol Invasions 11: 21-45. doi:10.1007/s10530-008-9318-y.

[3] Anagnostakis SL (1987) Chestnut Blight - the classical problem of an introduced pathogen. Mycologia 79: 23-37. doi:10.2307/3807741.

[4] Normile D (2008) Driven to extinction. Science 319: 1606-1609. doi:10.1126/science.319.5870.1606. PubMed: 18356500. 18356500 10.1126/science.319.5870.1606

[5] Elton CS (1958) The Ecology of Invasions by Animals and Plants. Chicago, IL, USA: Chicago University Press. 181pp.

[6] Williamson M (1996) Biological Invasions. London, UK: Chapman & Hall. 244pp.

[7] Davis MA (2009) Invasion Biology. Oxford, UK: Oxford University Press. 244pp.

[8] Pimentel D, Lach L, Zuniga R, Morrison D (2000) Environmental and economic costs of nonindigenous species in the United States. BioScience 50: 53-65.

[9] Simberloff D (2005) Non-native species do threaten the natural environment! J Agric Environ Ethics 18: 595-607. doi:10.1007/s10806-005-2851-0.

[10] Roy HE, Adriaens T, Isaac NJB, Kenis M, Onkelinx T et al. (2012) Invasive alien predator causes rapid declines of native European ladybirds. Divers Distrib 18: 717-725. doi:10.1111/j.1472-4642.2012.00883.x.

[11] Maron JL, Vila M (2001) When do herbivores affect plant invasion? Evidence for the natural enemies and biotic resistance hypotheses. Oikos 95: 361-373. doi:10.1034/j.1600-0706.2001.950301.x.

[12] Mitchell CE, Power AG (2003) Release of invasive plants from fungal and viral pathogens. Nature 421: 625-627. doi:10.1038/nature01317. PubMed: 12571594. 12571594 10.1038/nature01317

[13] Tilman D (2004) Niche tradeoffs, neutrality, and community structure: A stochastic theory of resource competition, invasion, and community assembly. Proc Natl Acad Sci U S A 101: 10854-10861. doi:10.1073/pnas.0403458101. PubMed: 15243158. 15243158 10.1073/pnas.0403458101 PMC503710

[14] Eisenhauer N, Fisichelli NA, Frelich LE, Reich PB (2012) Interactive effects of global warming and 'global worming' on the initial establishment of native and exotic herbaceous plant species. Oikos 121: 1121-1133. doi:10.1111/j.1600-0706.2011.19807.x.

[15] Webber BL, Scott JK (2012) Rapid global change: implications for defining natives and aliens. Glob Ecol Biogeogr 21: 305-311. doi:10.1111/j.1466-8238.2011.00684.x.

[16] Smith AL, Hewitt N, Klenk N, Bazely DR, Yan N et al. (2012) Effects of climate change on the distribution of invasive alien species in Canada: a knowledge synthesis of range change projections in a warming world. Environ Rev 20: 1-16. doi:10.1139/a11-020.

[17] Kurz WA, Dymond CC, Stinson G, Rampley GJ, Neilson ET et al. (2008) Mountain pine beetle and forest carbon feedback to climate change. Nature 452: 987-990. doi:10.1038/nature06777. PubMed: 18432244. 18432244 10.1038/nature06777

[18] Ragsdale DW, Landis DA, Brodeur J, Heimpel GE, Desneux N (2011) Ecology and management of soybean aphid in North America. Annu Rev Entomol 56: 375-399. doi:10.1146/annurev-ento-120709-144755. PubMed: 20868277. 20868277 10.1146/annurev-ento-120709-144755

[19] Heimpel GE, Ragsdale DW, Venette R, Hopper KR, O’Neil RJ et al. (2004) Prospects for importation biological control of the soybean aphid: anticipating potential costs and benefits. Ann Entomol Soc Am 97: 249-258. doi:10.1603/0013-8746(2004)097[0249:PFIBCO]2.0.CO;2.

[20] Hill CB, Chirumamilla A, Hartman GL (2012) Resistance and virulence in the soybean-*Aphis glycines* interaction. Euphytica 186: 635-646. doi:10.1007/s10681-012-0695-z.

[21] Pretty J (2008) Agricultural sustainability: concepts, principles and evidence. Philos Trans R Soc Lond B Biol Sci 363: 447-465. doi:10.1098/rstb.2007.2163. PubMed: 17652074. 17652074 10.1098/rstb.2007.2163 PMC2610163

[22] Pretty JN, Ball AS, Li XY, Ravindranath NH (2002) The role of sustainable agriculture and renewable-resource management in reducing greenhouse-gas emissions and increasing sinks in China and India. Philos Trans R Soc Lond A Math Phys Eng Sci 360: 1741-1761. doi:10.1098/rsta.2002.1029. PubMed: 12460495. 10.1098/rsta.2002.1029 12460495

[23] Helsel ZR (2006) Energy in pesticide production and use. In: Pimentel D Encyclopedia of Pest Management. London, UK: Taylor & Francis pp. 1-4.

[24] Wang M, Wu M, Huo H (2007) Life-cycle energy and greenhouse gas emission impacts of different corn ethanol plant types. Environ Res Lett 2: 024001. doi:10.1088/1748-9326/2/2/024001.

[25] Green MB (1987) Energy in pesticide manufacture. In: Helsel ZR Energy in Plant Nutrition and Pest Control Volume 2 Amsterdam, The Netherlands: Elsevier pp. 165-178.

[26] Ragsdale DW, McCornack BP, Venette RC, Potter BD, Macrae IV et al. (2007) Economic threshold for soybean aphid (Hemiptera : Aphididae). J Econ Entomol 100: 1258-1267. doi:10.1603/0022-0493(2007)100[1258:ETFSAH]2.0.CO;2. PubMed: 17849878. 17849878 10.1603/0022-0493(2007)100[1258:ETFSAH]2.0.CO;2

[27] Song F, Swinton SM (2009) Returns to integrated pest management research and outreach for soybean aphid. J Econ Entomol 102: 2116-2125. doi:10.1603/029.102.0615. PubMed: 20069840. 20069840 10.1603/029.102.0615

[28] Desneux N, O’Neil RJ, Yoo HJS (2006) Suppression of population growth of the soybean aphid, *Aphis glycines* Matsumura, by predators: the identification of a key predator and the effects of prey dispersion, predator abundance, and temperature. Environ Entomol 35: 1342-1349. doi:10.1603/0046-225X(2006)35[1342:SOPGOT]2.0.CO;2.

[29] Costamagna AC, Landis DA, Difonzo CD (2007) Suppression of soybean aphid by generalist predators results in a trophic cascade in soybeans. Ecol Appl 17: 441-451. doi:10.1890/06-0284. PubMed: 17489251. 17489251 10.1890/06-0284

[30] Gardiner MM, Landis DA, Gratton C, Difonzo CD, O’Neal ME et al. (2009) Landscape diversity enhances biological control of an introduced crop pest in the north-central USA. Ecol Appl 19: 143-154. doi:10.1890/07-1265.1. PubMed: 19323179. 19323179 10.1890/07-1265.1

[31] Woltz JM, Landis DA (2013) Coccinellid immigration to infested host patches influences suppression of *Aphis glycines* in soybean. Biol Contr 64: 330-337. doi:10.1016/j.biocontrol.2012.11.012.

[32] Bauer ME, Boethel DJ, Boyd ML, Bowers GR, Way MO et al. (2000) Arthropod populations in early soybean production systems in the mid-south. Environ Entomol 29: 312-328. doi:10.1603/0046-225X(2000)029[0312:APIESP]2.0.CO;2.

[33] Smith JF, Luttrell RG, Greene JK (2009) Seasonal abundance, species composition, and population dynamics of stink bugs in production fields of early and late soybean in south Arkansas. J Econ Entomol 102: 229-236. doi:10.1603/029.102.0132. PubMed: 19253641. 19253641 10.1603/029.102.0132

[34] Brown SA, Davis JA, Richter AR (2012) Efficacy of four insecticides on eggs of Nezara viridula (Hemiptera: Pentatomidae). Florida Entomol 95: 1182-1186. doi:10.1653/024.095.0449.

[35] Thrasher JD, Heuser G, Broughton A (2002) Immunological abnormalities in humans chronically exposed to chlorpyrifos. Arch Environ Health 57: 181-187. doi:10.1080/00039890209602934. PubMed: 12507170. 12507170 10.1080/00039890209602934

[36] Desneux N, Decourtye A, Delpuech JM (2007) The sublethal effects of pesticides on beneficial arthropods. Annu Rev Entomol 52: 81-106. doi:10.1146/annurev.ento.52.110405.091440. PubMed: 16842032. 16842032 10.1146/annurev.ento.52.110405.091440

[37] Floyd EY, Geist JP, Werner I (2008) Acute, sublethal exposure to a pyrethroid insecticide alters behavior, growth, and predation risk in larvae of the fathead minnow (*Pimephales promelas*). Environ Toxicol Chem 27: 1780-1787. doi:10.1897/07-448.1. PubMed: 18380520. 18380520 10.1897/07-448.1

[38] McCornack B, Ragsdale DW (2006) Efficacy of thiamethoxam to suppress soybean aphid populations in Minnesota soybean. Crops Manage Pp. doi:10.1094/CM-2006-0915-1001-RS.

[39] Magalhaes LC, Hunt TE, Siegfried BD (2009) Efficacy of neonicotinoid seed treatments to reduce soybean aphid populations under field and controlled conditions in Nebraska. J Econ Entomol 102: 187-195. doi:10.1603/029.102.0127. PubMed: 19253636. 19253636 10.1603/029.102.0127

[40] Heuer E, Flake M (2000) Environmental impact assessment of pesticide use within the framework of Life Cycle Assessment. Z Fur Pflanzenkr Pflanzenschutzj Plants Dis Protect: 735-744.

[41] Alluvione F, Moretti B, Sacco D, Grignani C (2011) EUE (energy use efficiency) of cropping systems for a sustainable agriculture. Energy 36: 4468-4481. doi:10.1016/j.energy.2011.03.075.

[42] Bartlett MD, James IT (2011) A model of greenhouse gas emissions from the management of turf on two golf courses. Sci Total Environ 409: 1357-1367. doi:10.1016/j.scitotenv.2010.12.041. PubMed: 21288561. 21288561 10.1016/j.scitotenv.2010.12.041

[43] Pedigo LP, Rice M (2006) Entomology and Pest Management. Upper Saddle River, New Jersey, USA. Pearson : Prentice Hall . 749pp

[44] Yu SJ (2008) The Toxicology and Biochemistry of Insecticides. Boca Raton, FL, USA: CRC Press. 276pp.

[45] Solomon S, Qin D, Alley RB, Berntsen NL, Bindoff Z et al. (2007) Climate Change 2007: The Physical Science Basis. Contribution of Working Group I to the Fourth Assessment Report of the Intergovernmental Panel on Climate Change. Cambridge, UK: Cambridge University Press.

[46] Ware GW, Whitacre DM (2004) The Pesticide Book, 6th Edition. Willoughby, OH, USA: MeisterPro Information Resources

[47] Wang MQ (1999) GREET Transportation Fuel Cycle Model Volume 1: Methodology, Development, Use, and Results. Center for Transportation Research, Energy Systems Division, Argonne Lational Laboratory . p. 1. p. 5

[48] Olson K, Badibanga T, DiFonzo C (2008) Famer’s awareness and use of IPM for soybean aphid control: Report of survey results for the 2004, 2005, 2006, and 2007 crop years. St. Paul, MN, USA: University of Minnesota.

[49] Grisso R, Perumpral JV, Vaughan D, Robertson GT, Pitman R (2010) Predicting tractor diesel fuel consumption. Blacksburg, VA, USA: Virginia Polytechnic Institute and State University.

[50] Hodgson EW, McCornack BP, Tilmon K, Knodel J (2012) Management recommendations for soybean aphid (Hemiptera: Aphididae) in the United States. J Integrated Pests Manage 3.

[51] Woltz JM, Isaacs R, Landis DA (2012) Landscape structure and habitat management differentially influence insect natural enemies in an agricultural landscape. Agric Ecosyst Environ 152: 40-49. doi:10.1016/j.agee.2012.02.008.

[52] Landis DA, Gardiner MM, van der Werf W, Swinton SM (2008) Increasing corn for biofuel production reduces biocontrol services in agricultural landscapes. Proc Natl Acad Sci U S A 105: 20552-20557. doi:10.1073/pnas.0804951106. PubMed: 19075234. 19075234 10.1073/pnas.0804951106 PMC2603255

[53] Costanza R, d’Arge R, de Groot R, Farber S, Grasso M et al. (1997) The value of the world’s ecosystem services and natural capital. Nature 387: 253-260. doi:10.1038/387253a0.

[54] Gurr GM, Wratten SD, editors (2000). iol Control Measures Success Dordrecht Kluwer: 429.

[55] Heinz KM, van Driesche R, Parrella MP, editors (2005) Biocontrol in Protected Culture. Ball Publishing.

[56] Losey JE, Vaughan M (2006) The economic value of ecological services provided by insects. BioScience 56: 311-323. PubMed: 19831280

[57] Van Driesche R, Hoddle M, Center TD (2008) Control of Pests and Weeds by Natural Enemies: An Introduction to Biological Control. Wiley-Blackwell.

[58] Hodgson EW, Kemis M, Geisinger B (2012) Assessment of Iowa soybean growers for insect pest management practices. J Extension.

[59] Johnson KD, O’Neal ME, Ragsdale DW, Difonzo CD, Swinton SM et al. (2009) Probability of cost-effective management of soybean aphid (Hemiptera: Aphididae) in North America. J Econ Entomol 102: 2101-2108. doi:10.1603/029.102.0613. PubMed: 20069838. 20069838 10.1603/029.102.0613

[60] Rhainds M, Yoo HJS, Kindlmann P, Voegtlin D, Castillo D et al. (2010) Two-year oscillation cycle in abundance of soybean aphid (Hemiptera: Aphididae) in Indiana. Agric Forest Entomol 12: 251-257.

[61] Gardiner M, Landis DA, Gratton C, Schmidt N, O’Neal M et al. (2010) Landscape composition influences the activity density of Carabidae and Arachnida in soybean fields. Biol Contr 55: 11-19. doi:10.1016/j.biocontrol.2010.06.008.

[62] Noma T, Gratton C, Colunga-Garcia M, Brewer MJ, Mueller EE et al. (2010) Relationship of soybean aphid (Hemiptera: Aphididae) to soybean plant nutrients, landscape structure, and natural enemies. Environ Entomol 39: 31-41. doi:10.1603/EN09073. PubMed: 20146837. 20146837 10.1603/EN09073

[63] Schmidt NP, O’Neal ME, Singer JW (2007) Alfalfa living mulch advances biological control of soybean aphid. Environ Entomol 36: 416-424. doi:10.1603/0046-225X(2007)36[416:ALMABC]2.0.CO;2. PubMed: 17445377. 17445377 10.1603/0046-225x(2007)36[416:almabc]2.0.co;2

[64] Koch RL, Porter PM, Harbur MM, Abrahamson MD, Wyckhuys KAG et al. (2012) Response of soybean insects to an autumn-seeded rye cover crop. Environ Entomol 41: 750-760. doi:10.1603/EN11168.

[65] Lundgren JG, Hesler LS, Clay SA, Fausti SF (2013) Insect communities in soybeans of eastern South Dakota: The effects of vegetation management and pesticides on soybean aphids, bean leaf beetles, and their natural enemies. Crop Protect 43: 104-118. doi:10.1016/j.cropro.2012.08.005.

[66] Asplen MK, Wyckhuys KAG, Heimpel GE (2011) Parasitism of autumnal morphs of the soybean aphid, *Aphis glycines* (Hemiptera: Aphididae), by *Binodoxys communis* (Hymenoptera: Braconidae) on buckthorn. Ann Entomol Soc Am 104: 935-944. doi:10.1603/AN10172.

[67] Heimpel GE, Frelich LE, Landis DA, Hopper KR, Hoelmer KA et al. (2010) European buckthorn and Asian soybean aphid as components of an extensive invasional meltdown in North America. Biol Invasions 12: 2913-2931. doi:10.1007/s10530-010-9736-5.

[68] Riedell WE, Catangui MA, Beckendorf EA (2009) Nitrogen fixation, ureide, and nitrate accumulation responses to soybean aphid injury in *Glycine max* . J Plant Nutr 32: 1674-1686. doi:10.1080/01904160903150925.

